# Supplementary material for: Self- versus clinician-collected swabs in anal cancer screening: A clinical trial
Source: PLoS One. 2025 Jan 9;20(1):e0312781. doi: 10.1371/journal.pone.0312781 (PMC11717180; doi:10.1371/journal.pone.0312781)
Supplement: S1 Table — (DOCX) [file pone.0312781.s001.docx]

***S1 Table: Comparison between self-collected and clinician-collected anal swabs in cytological assessment***

| **Cytology results** | **Total (N=100)**  n (%, 95% CI) | | | **SCA first (N=54)**  n (%, 95% CI) | | | **CCA first (N=46)**  n (%, 95% CI) | | |
| --- | --- | --- | --- | --- | --- | --- | --- | --- | --- |
|  | **SC** | **CC** | *P-value* | **SC** | **CC** | *P-value* | **SC** | **CC** | *P-value* |
| **Sample adequacy**  Satisfactory | 77 (**77.0**, 67.6–84.3) | 84 (**84.0**, 75.3–90.0) | 0.162 | 45 (**83.3**, 70.6–91.2) | 47 (**87.0**, 74.9–93.8) | 0.564 | 32 (**69.6**, 54.5–81.3) | 37 (**80.4**, 66.0–89.7) | 0.166 |
| **Transformation zone**  Detected | 58 (**58.0**,48.0–67.4) | 54 (**54.0**, 44.1–63.6) | 0.480 | 36 (**66.7**, 52.9–78.1) | 35 (**64.8**, 51.0–76.5) | 0.819 | 22 (**47.8**, 33.6–62.4) | 19 (**41.3**, 27.8–56.3) | 0.405 |
| **Identified abnormalities***  HSIL  ASC-H  HSIL/ASC-H  ASC-US  LSIL  LSIL/ASC-US  Any abnormality | 14 (**20.6**, 12.5–32.1)  12 (**17.6**, 10.2–28.8)  26 (**38.2**, 27.3–50.5)  11 (**16.2**, 9.1–27.1)  17 (**25.00**, 16.0–36.8)  28 (**41.2**, 30.0–53.4)  54 (**79.4**, 67.9–87.5) | 8 (**11.8**, 5.9–22.0)  12 (**17.7**, 10.2–28.8)  20 (**29.4**, 19.7–41.5)  10 (**14.7**, 8.0–25.5)  26 (**38.2**, 27.3–50.5)  36 (**52.9**, 40.9–64.6)  56 (**82.4**, 71.2–89.8) | 0.134  0.088  0.480 | 8 (**20.0**, 10.1–35.7)  7 (**17.5**, 8.4–33.0)  15 (**37.5**, 23.7–53.7)  6 (**15.0**, 6.7–30.2)  10 (**25.0**, 13.7–41.1)  16 (**40.0**, 25.8–56.2)  31 (**77.5**, 61.6–88.1) | 3 (**7.5**, 2.4–21.4)  7 (**17.5**, 8.4–33.0)  10 (**25.0**, 13.7–41.1)  7 (**17.5**, 8.4–33.0)  15 (**37.5**, 23.7–53.7)  22 (**55.0**, 39.1–69.9)  32 (**80.0**, 64.3–89.9) | 0.132  0.109  0.655 | 6 (**21.4**, 9.6–41.2)  5 (**17.9**, 7.3–37.4)  11 (**39.3**, 22.6–58.9)  5 (**17.9**, 7.3–37.4)  7 (**25.0**, 12.0–44.9)  12 (**42.9**, 25.5–62.1)  23 (**82.1**, 62.6–92.7) | 5 (**17.9**, 7.3–37.4)  5 (**17.9**, 7.3–37.4)  10 (**35.7**, 19.8–55.5)  3 (**10.7**, 3.3–29.6)  11 (**39.3**, 22.6–58.9)  14 (**50.0**, 31.5–68.5)  24 (**85.7**, 66.5–94.8) | 0.655  0.480  0.564 |

*Excludes samples that were classed as “unsatisfactory” for sample adequacy by either SCA or CCA or both (denominator = 68 (40 SCA first; 28 for CCA first). HSIL: High-grade squamous intraepithelial lesion; ASC-H: Atypical squamous cells, cannot exclude high-grade squamous intraepithelial lesion; ASC-US: Atypical squamous cells of uncertain significance; LSIL: Low-grade squamous intraepithelial lesion. P values are from McNemar paired case-control study test.
