# Supplementary material for: Self- versus clinician-collected swabs in anal cancer screening: A clinical trial
Source: PLoS One. 2025 Jan 9;20(1):e0312781. doi: 10.1371/journal.pone.0312781 (PMC11717180; doi:10.1371/journal.pone.0312781)
Supplement: S2 Table — (DOCX) [file pone.0312781.s002.docx]

***S2 Table: Comparison between self-collected and clinician-collected swabs in HPV DNA testing***

| **HPV result** | **Total (N=100)**  n (%, 95% CI) | | | **SCA first (N=54)**  n (%, 95% CI) | | | **CCA first (N=46)**  n (%, 95% CI) | | |
| --- | --- | --- | --- | --- | --- | --- | --- | --- | --- |
|  | **SCA** | **CCA** | *P-value* | **SCA** | **CCA** | *P-value* | **SCA** | **CCA** | *P-value* |
| **Sample validity***  Valid | 94 (**94.0**, 87.2–97.3) | 95 (**95.0**, 88.4–97.9) | 0.564 | 52 (**96.3**, 86.0–99.1) | 54 (**100.0**, -) | 0.157 | 42 (**91.3**, 78.5–96.8) | 41 (**89.1**, 76.0–95.5) | 0.317 |
| **HPV present****  Any HR-HPV  HPV16  HPV18  Other HR-HPV | 63 (**67.7**, 57.5–76.5)  32 (**34.4**, 25.4–44.7)  9 (**9.7**, 5.1–17.7)  48 (**51.6**, 41.4–61.7) | 66 (**71.0**, 60.8–79.4)  28 (**30.1**, 21.6–40.3)  8 (**8.6**, 4.3–16.4)  54 (**58.1**, 47.7–67.8) | 0.317  0.103  0.564  0.083 | 39 (**75.0**, 61.2–85.1)  20 (**38.5**, 26.1–52.6)  4 (**7.7**, 2.8–19.2)  29 (**55.8**, 41.9–68.8) | 41 (**78.8**, 65.3–88.1)  18 (**34.6**, 22.8–48.7)  3 (**5.8**, 1.8–16.8)  31 (**59.6**, 45.6–72.2) | 0.414  0.317  0.317  0.414 | 24 (**58.5**, 42.7–72.8)  12 (**29.3**, 17.1–45.3)  5 (**12.2**, 5.0–26.7)  19 (**46.3**, 31.4–61.9) | 25 (**61.0**, 45.0–74.9)  10 (**24.4**, 13.4–40.2)  5 (**12.2**, 5.0–26.7)  23 (**56.1**, 40.4–70.7) | 0.564  0.157  1.000  0.103 |

*Valid test for *all* HPV testing; **Only includes participants where both SCA and CCA tests were valid for a particular HPV subtype (denominator = 93 (52 SCA first; 41 for CCA first); †Non HPV16/18 HR genotype.
