# Supplementary figures and images for: Self- versus clinician-collected swabs in anal cancer screening: A clinical trial
Source: PLoS One. 2025 Jan 9;20(1):e0312781. doi: 10.1371/journal.pone.0312781 (PMC11717180; doi:10.1371/journal.pone.0312781)

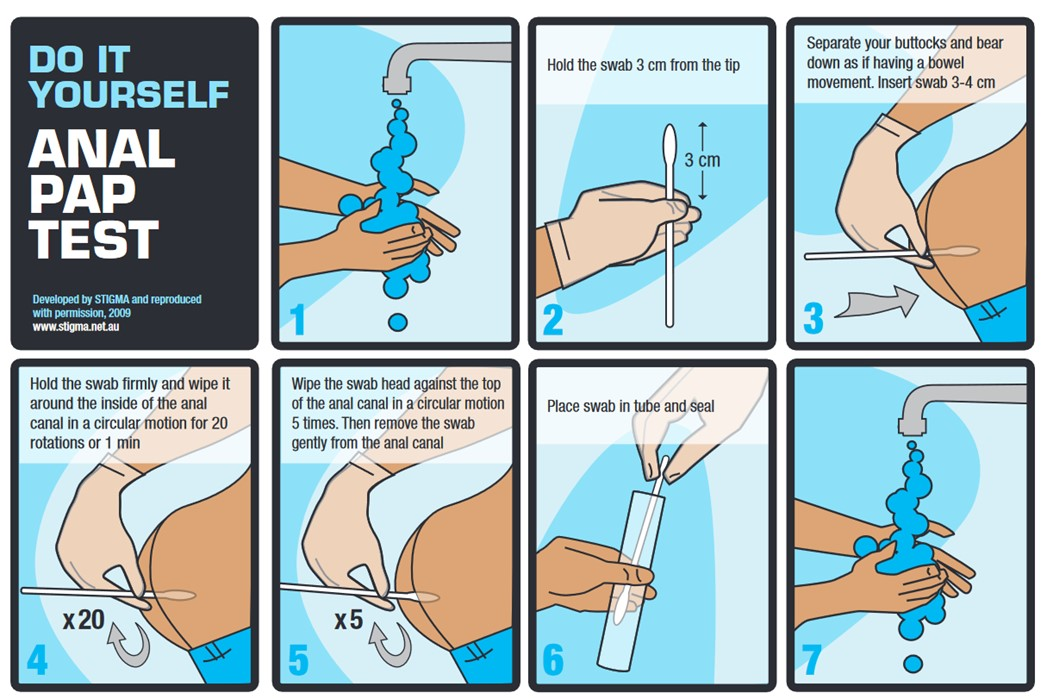

Supplement: S1 Fig — (TIF) [file pone.0312781.s003.tif]
