# Supplementary material for: Self- versus clinician-collected swabs in anal cancer screening: A clinical trial
Source: PLoS One. 2025 Jan 9;20(1):e0312781. doi: 10.1371/journal.pone.0312781 (PMC11717180; doi:10.1371/journal.pone.0312781)
Supplement: S2 Fig — (PDF) [file pone.0312781.s004.pdf]

Study Number			

2x2			

TICAT QUESTIONNAIRE 1 - ACCEPTABILITY OF SCREENING TESTS

Thank you for participating in the Acceptability component of the TICAT study.

This will help us to assess the impact of the self-collected swab versus the clinician-collected swab on your wellbeing. It will give us important information about the different types of reactions that can occur as a result of these screening methods. We will use this information to provide information and support to help people such as yourself in the future.

The questionnaire takes about 5 minutes to complete.

Please complete all questions. There are no right or wrong answers. Your time and effort is appreciated.

If you have any questions about this survey please phone contact the Study Nurse or Doctor

We would like to know how you found the anal swabs. Please indicate the response which most closely applies to you.

First, please tell us how comfortable / uncomfortable each examination was:

	Not at all	A little	A fair bit	Quite a lot	Very much
Did you find the anal swab **you inserted yourself** uncomfortable?	☐	☐	☐	☐	☐
Did you find the anal swab **that was inserted by the nurse/doctor** uncomfortable?	☐	☐	☐	☐	☐
**Now tell us whether these examinations/ tests were painful or not**					
Did the anal swab **you inserted yourself** hurt?	☐	☐	☐	☐	☐
Did the anal swab **that was inserted by the nurse/doctor** hurt?	☐	☐	☐	☐	☐
**How did you feel during the tests**					
Did you feel relaxed collecting your own anal swab?	☐	☐	☐	☐	☐
	**Very Dissatisfied**	**Dissatisfied**	**Satisfied**	**Very satisfied**	
Were you satisfied with the instructions given to you before you collected your anal swab?	☐	☐	☐	☐	
Overall, what was your preference between the self-collected anal swab and the clinician collected anal swab?	I much preferred the self-collected swab	I partly preferred the self-collected swab	I have no preference	I partly preferred the clinician-collected swab	I much preferred the clinician-collected swab
Why did you have this preference?					
Do you have any other comments you would like to add?					

This is the end of the questionnaire.

Thank you for your time.
