# Supplementary material for: Self- versus clinician-collected swabs in anal cancer screening: A clinical trial
Source: PLoS One. 2025 Jan 9;20(1):e0312781. doi: 10.1371/journal.pone.0312781 (PMC11717180; doi:10.1371/journal.pone.0312781)
Supplement: Attachment — Submitted filename: Response letter to reviewers_v1.0.pdf [file pone.0312781.s005.pdf]

19 August 2024

Gulzhanat Aimagambetova
Academic Editor
PLOS ONE

Dear Dr Aimagambetova,

Re: PONE-D-24-14018 – Self- Versus Clinician-Collected Swabs in Anal Cancer Screening: A Clinical Trial

We are pleased to resubmit our revised original research paper, "*Self- Versus Clinician-Collected Swabs in Anal Cancer Screening: A Clinical Trial*", for consideration for publication in PLOS ONE.

We have worked to address your comments, and those of the reviewers, and provide our responses overleaf. The line references refer to the track-change version of the revised manuscript.

Regarding the funding statement, please amend online to:

"We would like to thank the St Vincent's Clinic Foundation for funding this project. The funders had no role in study design, data collection and analysis, decision to publish, or preparation of the manuscript."

Please do not hesitate to contact us should you have any queries or further comments.

Yours sincerely,

Professor Richard J. Hillman

Editorial comments

---

1. **In addition to the reviewers' comments, please perform editing of the discussion as recommended:**

   Thank you for the editing recommendation. We have now edited the discussion section, following the PLOS ONE suggested structure.

2. **When submitting your revision, please ensure that your manuscript meets PLOS ONE's style requirements, including those for file naming.**

   Apologies for this oversight. All files are now named according to the PLOS ONE style requirements.

3. **Thank you for stating the following financial disclosure: "We would like to thank the St Vincent's Clinic Foundation for funding this project." Please state what role the funders took in the study. If the funders had no role, please state: "The funders had no role in study design, data collection and analysis, decision to publish, or preparation of the manuscript." Please include this amended Role of Funder statement in your cover letter; we will change the online submission form on your behalf.**

   Please amend the financial disclosure statement to: *"We would like to thank the St Vincent's Clinic Foundation for funding this project. The funders had no role in study design, data collection and analysis, decision to publish, or preparation of the manuscript."*

4. **Please remove any funding-related text from the manuscript and let us know how you would like to update your Funding Statement. Currently, your Funding Statement reads as follows: "We would like to thank the St Vincent's Clinic Foundation for funding this project." Please include your amended statements within your cover letter; we will change the online submission form on your behalf.**

   We don't believe there is any mention of funding in the text. However, please amend the financial disclosure statement to: *"We would like to thank the St Vincent's Clinic Foundation for funding this project. The funders had no role in study design, data collection and analysis, decision to publish, or preparation of the manuscript."*

5. **Please include captions for your Supporting Information files at the end of your manuscript, and update any in-text citations to match accordingly. Please see our Supporting Information guidelines for more information: http://journals.plos.org/plosone/s/supporting-information.**

   Apologies for this oversight. We have now added in the captions for the supporting information files at the end of the manuscript and updated the in-text citations to match accordingly, and according to the journal guidelines.

Reviewer 1 comments

1. **Sample size: could the authors explain how to calculate the sample size? The small sample used could result in limited study power as the author acknowledges in the limitations. On the other hand, there is a great heterogenicity of participants, in terms of sex and HIV serostatus. Both variables influence the prevalence of HPV and cytological abnormalities and should be analysed separately. Could the authors explain how heterogenicity could be addressed; perhaps women and heterosexual men could be excluded or analysed separately?**

   Thank you very much for raising this important point. As is indicated in the methods section, the participants in this study were essentially a convenience sample from a busy anal dysplasia clinic. We acknowledge in the discussion section that the small sample size did limit the power of the study.

   At the time of the design of the study, there were no data upon which to base a formal sample size calculation. However, the sample size, whilst limited, did allow us to gain valuable insights into the comparison between the two sampling methods. We have added an additional sentence in the discussion section to reflect this as follows:

   Lines 311–312: *However, despite the small sample size, we were still able to gain valuable insights into the comparison between SCA and CCA sampling methods.*

   Regarding your second point, we wanted to represent routine clinical practice, which would include the great heterogenicity mentioned. To analyse each subgroup along the lines suggested would require a much larger sample size, which was unfortunately beyond the scope of this project.

2. **The authors explain in methods that participants underwent a high resolution anoscopy. Why did the authors not use HRA as the gold standard for cytology accuracy (sensitivity and specificity) of the two swabs? I suggest adding this limitation in case authors are unable to performs the aforementioned analysis.**

   Thank you very much for this important suggestion. We did undertake an analysis using HRA to determine the sensitivity and specificity of both sample methods; however, the small sample size led to difficulties interpreting these data.

   Furthermore, together with our co-authors, we felt that it was more important to compare swab with swab in this paper given our focus on whether a SCA performs as well as a CCA, which is why we chose to focus on CCA as the gold standard (rather than comparing both with histology results).

3. **Agreement between swabs is good, except for adequate cytology sample, which may be associated with decreased HSIL/ASC-H sensitivity. What do the authors think about this topic? Could the authors add this information in the text and give their opinion.**

   This is an important point. We have modified Table 5 to remove sensitivity and specificity values for sample adequacy/validity, since it is not appropriate to determine these values in this context. We have left in concordance values, which are useful to compare sample adequacy between swab collection methods.

4. **Table 3A: I suggest using the same words as in the text: Swab adequate to cellular adequacy; Non-negative diagnosis to Any cytological abnormality.**

   Thank you for this suggestion, which we have implemented.

Reviewer 2 comments

1. **Table 5 – Results' concordance should include not only in percentages but also the denominators/numbers used. Authors should also clarify in the Results how they assessed Sensitivity and Specificity as this was not described in the text nor in the legend, particularly as anal histology was not available to define "diseased" and "non-diseased" individuals for clinical sensitivity and specificity assessment.**

   Thank you for noticing that we had not made mention of sensitivity and specificity in the main manuscript text. We have now added in a paragraph on these results. However, we do state that clinician-collected results were used as the gold standard in this comparison. The rationale being that this paper is comparing self- with clinician-collected swab results, even though we do acknowledge that in the field of anal cancer, an HRA-biopsy/histology is considered the gold standard for anal cancer diagnosis.

   As you suggest, we have also added in the numerator/denominator for the concordance calculations to make this clearer for the reader.

2. **Several tables in the manuscript report overall results of self-collected and clinician-collected samples as well as separately according to the order of collection. As no statistical difference was found in the results based on the order of collection, as shown in Table 3, the authors should simplify Tables 2, 4 and 5 by reporting only the overall test results on SCA and CCA. Additional information on collection order could be included as supplementary tables.**

   Thank you for this suggestion. We agree it makes tables 2 and 4 clearer and easier to read and have removed the data on different collection orders. We have moved the additional information into supplementary, since it is a unique part of this paper that swab order was investigated so thoroughly and that we reported results based on swab order.

   However, we have not made any further changes to Table 5, since this table is not showing data based on swab collection order.

3. **"Discussion" page 15 lines 242–249: The authors, in comparing their results of sample adequacy of SCA and CCA with those reported in previously published studies should also include the potential role of different "sample collection devices" used in the previous reports. Different preanalytical and analytical workflows must be considered when comparing the results of different studies.**

   Thank you for this suggestion. We have added in a section discussing the importance of the sample collection device as follows:

   Lines 286–290: *The sample collection device used across different studies may also be important in the context of optimising cellular adequacy. A FLOQSwab® flocked swab was used in this study, while the other three studies comparing SCA versus CCA for cytology testing used a Dacron swab. However, evidence suggest that flocked swabs may perform slightly better than Dacron swabs for anal cytology (26, 27).*

4. **“Data analysis” page 6 lines 129–130: Authors state that for cellular adequacy, unsatisfactory anal cytology samples and those of “low cellularity” were aggregated. It would be of value to report the number (%) of each of these two categories in the text and whether lower HPV positivity rates were observed in “low cellularity” samples.**

   We have made the methods section clearer as to the rationale for combining these two classifications as follows:

   Lines 133–138: *The Bethesda system notes that if a sample has low cellularity (and would usually be classified as ‘unsatisfactory’), but an abnormal cell(s) is seen in the sample, it is reclassified as ‘adequate’, and the abnormal result recorded. Since we wanted to compare true cellular adequacy (regardless of cytology results), for calculations relating to the cellular adequacy of the cytology slide, slides deemed ‘unsatisfactory’ and those ‘of low cellularity’ (but an abnormality was seen) were aggregated and denoted ‘unsatisfactory’ for the purposes of the analysis.*

   Since only a few samples were classified as ‘low cellularity’, it would not be possible to report the study findings separately.

5. **“Study participants” page 4: Reasons for patients’ referral to “routine appointments” at the DACS Clinic should be better clarified in this section.**

   These are standard follow-up appointments for people living with anal dysplasia in a context where routine treatment was not available.

   We have amended the wording to:

   Lines 75–77: *Eligible participants were patients attending a routine appointment for monitoring and management of their anal dysplasia at the Dysplasia and Anal Cancer Services (DACS) at St Vincent’s Hospital, Darlinghurst, Sydney, and undergoing a high resolution anoscopy (HRA).*

6. **The Discussion should underline that a larger study population would be required to assess diagnostic accuracy of cytology and/or HPV testing in SCA as compared to CCA, to determine relative clinical sensitivity and specificity.**

   Reviewer 1 raised a similar point, which we agree with, and have modified the text as indicated.
